# Limited effectiveness of radioiodine therapy for residual cervical lymph node metastases in radioiodine-naïve papillary thyroid cancer

**DOI:** 10.3389/fendo.2026.1838143

**Published:** 2026-07-06

**Authors:** Seyfettin Ilgan, M.Özdeş Emer, Ferit Taneri, Erdinç Aygenç, Serdar Özbaş, Murat Akın, Erkan Öztürk, Banu Bilezikçi, Seza A. Gulec

**Affiliations:** 1 Department of Nuclear Medicine, Güven Hospital, Ankara, Türkiye; 2 Department of Nuclear Medicine, Faculty of Medicine, Maltepe University, İstanbul, Türkiye; 3 Endocrine Surgery, Private Practice, Ankara, Türkiye; 4 Department of Head and Neck Surgery, Güven Hospital, Ankara, Türkiye; 5 Department of General Surgery, Faculty of Medicine, Gazi University, Ankara, Türkiye; 6 Department of Pathology, Güven Hospital, Ankara, Türkiye; 7 HCA Florida Healthcare, Aventura Hospital, Aventura, FL, United States; 8 Miami Cancer Research Center, Miami, FL, United States

**Keywords:** cervical lymph node metastasis, iodine avidity, papillary thyroid carcinoma, radioiodine therapy, residual nodal disease, treatment response

## Abstract

**Background:**

Radioiodine (RAI) therapy is frequently used to treat suspected or documented cervical lymph node metastases in papillary thyroid carcinoma (PTC), although its effectiveness for structurally documented residual nodal disease remains uncertain.

**Methods:**

This retrospective analysis of a prospectively followed cohort included RAI-naïve patients with PTC who had structurally confirmed cervical nodal metastases identified after surgery. Patients with documented metastatic lymph nodes in recently operated compartments, or those declining immediate reoperation, received upfront RAI therapy. Treatment response was evaluated at both lymph-node and patient levels using serial ultrasonography and biochemical follow-up.

**Results:**

A total of 146 cervical lymph node metastases in 71 patients were analyzed. Of these, 39 (26.7%) were RAI-avid, 89 (61%) were RAI-non-avid, and 18 (12.3%) were indeterminate due to remnant interference. Among RAI-avid nodes, complete and partial responses were observed in 38.5% and 20.5%, respectively. Across all nodes, complete and partial responses were seen in 10.3% and 6.2%, while no objective response occurred in 83.5%. RAI-non-avid disease was frequent among aggressive histologic subtypes; however, most non-avid metastases arose from tumors with otherwise indolent histology. At the patient level, a complete response was achieved in 11% (8/71). All responders remained in the excellent response category at a median follow-up of 77 months (range 32–156), indicating a durable treatment effect.

**Conclusion:**

RAI-non-avid nodal metastases were common and represented the primary determinant of treatment failure. Even among RAI-avid lesions, response rates were modest. These findings suggest that lesion-level RAI-avidity is the principal determinant of therapeutic benefit and support a more selective, biology-guided approach to RAI therapy in patients with residual nodal disease.

## Introduction

The 2025 American Thyroid Association (ATA) guidelines for the management of differentiated thyroid cancer introduced a revised four-tier risk stratification system for papillary thyroid carcinoma (PTC), classifying patients as low, low-intermediate, intermediate-high, and high risk (1). Although the ATA-2025 guidelines provide a more refined risk-stratification framework, several authors have highlighted both their strengths and limitations, particularly regarding management recommendations for intermediate-risk disease and integration of emerging molecular and functional imaging data (2, 3). Within this framework, there is broad consensus that radioiodine (RAI) therapy is not indicated for low-risk PTC patients and is generally recommended for high-risk patients. In contrast, its role in the intermediate-risk spectrum—largely comprising patients with cervical lymph node metastases and/or histopathologic features associated with an increased likelihood of nodal involvement, such as vascular invasion, multifocality, and aggressive histology—remains controversial and is left to multidisciplinary judgment (1). In this setting, RAI therapy is often administered with the intent of eradicating suspected but undocumented nodal metastases, rather than to treat structurally confirmed disease.

Given the indolent nature of PTC, demonstrating a meaningful clinical benefit for disease that is, by definition, unproven is inherently difficult; therefore, a more clinically relevant and testable question is whether RAI therapy is effective for known residual nodal metastases. Because nearly 90% of residual disease after initial therapy is attributable to nodal involvement (4), addressing this question is critical for the appropriate use of RAI therapy in patients with suspected or documented nodal disease—a population that largely overlaps with the intermediate-risk group—where controversy persists.

Several retrospective series have reported high response rates for RAI-avid lymph node metastases (76-96%), with apparent efficacy comparable to or exceeding that of reoperative surgery (65-75%) (5–14). However, large-scale studies have failed to demonstrate a clear reduction in recurrence rates with RAI therapy in low- and intermediate-risk patients and have reported substantial rates of persistent biochemical (14-22%) and structural disease (21-34%) following empiric administration of high RAI activities (15–20). Persistent disease has also been observed in 52% of initially N1 patients one year after empiric administration of high RAI-activity (5550 MBq), a dose generally considered sufficient for nodal disease treatment (21).

Consequently, it remains unclear whether RAI therapy meaningfully reduces persistent nodal disease or whether its benefit for nodal disease is more limited than widely believed. The routine prioritization of reoperative surgical excision of documented structural nodal disease, followed by selective use of RAI therapy, further complicates evaluation of its independent therapeutic effect on metastatic lymph nodes, as this sequence obscures whether treatment is given to patients already rendered disease-free or whether adjuvant RAI therapy is eliminating residual—but clinically undocumented—nodal metastases.

Existing discussions of residual cervical nodal disease have largely focused on structural persistence identified after initial RAI administration, with limited guidance for RAI-naïve patients. Notably, the ATA-2025 guidelines neither discourage RAI use nor provide evidence-based recommendations regarding its effectiveness in this setting.

Administering RAI therapy for documented nodal metastases in RAI-naïve patients may offer two potential advantages over a surgery-first strategy. First, the presence of residual nodal disease enables direct assessment of tumor RAI-avidity and may prevent repeated empirical RAI therapy after future reoperations for RAI-non-avid disease. Second, common surgical practice is to defer reoperations within the same surgical compartment for several months to reduce the increased operative risks associated with tissue inflammation, adhesions, edema, and scar formation (22). Resolution of postoperative inflammatory changes during this interval is also critical for improving the diagnostic accuracy of imaging modalities used in comprehensive nodal mapping and surgical planning, thereby reducing the risk of incomplete resection and the need for repeat surgical intervention. Because RAI therapy remains relevant within a surgery-first paradigm, upfront treatment in selected patients during this interval may eradicate RAI-avid nodal metastases, potentially obviating the need for higher-risk reoperations, while also facilitating follow-up through ablation of residual thyroid tissue. The successful eradication of RAI-avid nodal disease may also reduce healthcare costs associated with reoperative surgery, hospitalization, and perioperative management.

Despite residual or suspected nodal disease remaining a principal indication for RAI therapy in intermediate-risk PTC, no prospective study has systematically evaluated its efficacy using documented pre-treatment nodal disease as the therapeutic target. To address this knowledge gap, follow-up data from a prospectively followed cohort of patients treated with RAI therapy for documented residual cervical metastatic lymph nodes under a predefined institutional protocol were retrospectively analyzed to assess the effectiveness of RAI for nodal disease.

## Materials and methods

### Study approval

This retrospective study was reviewed by the Güven Hospital Ethics Committee, which determined that formal ethical approval was not required due to the retrospective analysis of anonymized clinical data. The requirement for written informed consent was waived. This was a single-center study conducted at Güven Hospital. All surgical procedures, imaging evaluations, RAI administrations, and follow-up assessments were performed according to a single institutional protocol.

The study cohort was managed according to a predefined institutional clinical protocol developed for patients with structurally documented residual cervical lymph node metastases detected during early postoperative evaluation. Although patients were followed prospectively within routine clinical practice, the present investigation represents a retrospective analysis of these prospectively collected clinical and imaging data. The protocol was not conducted as a registered prospective clinical trial.

### Patients

**Figure 1:**
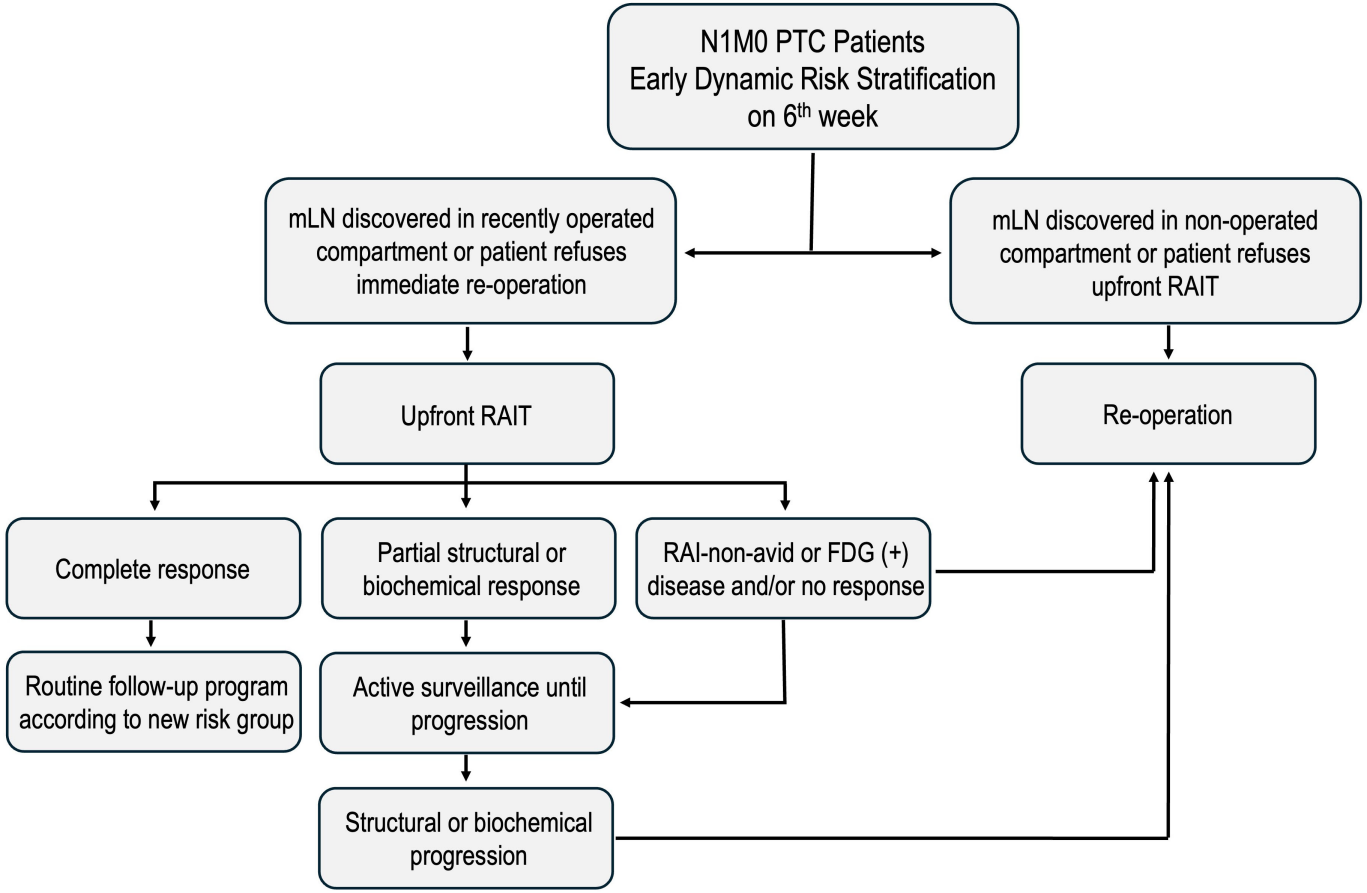
Institutional protocol for selecting RAI therapy versus reoperation in patients with residual metastatic lymph nodes identified during early dynamic risk stratification. Postoperative assessment includes neck US, thyroid function tests, Tg, and anti-Tg measurements as standard, with additional imaging performed as clinically indicated. mLN, metastatic lymph node.

The study cohort included 71 patients with PTC who underwent total thyroidectomy, with selective cervical lymph node dissection in 62 patients and without node dissection in 9 patients, between January 2011 and September 2024. All patients underwent comprehensive ultrasound (US) evaluation 6 weeks after the initial surgery, during which residual cervical lymph node metastases were identified. Metastatic disease was confirmed by fine-needle aspiration cytology and/or Thyroglobulin (Tg) washout or surgical histopathology in all cases. Patients with documented residual nodal metastases in the recently operated surgical compartment, or who declined immediate reoperation as the standard of care, were enrolled in this institutional protocol and treated with upfront RAI. All patients were RAI-naïve at the time of enrollment. The algorithm for selection of RAI versus reoperation is shown in **Figure 1**.

The study cohort consisted of PTC patients with lymph node metastases confined to the neck and without distant metastases who received RAI therapy for structurally documented residual cervical disease. Eligible lymph nodes were confined to the neck, measurable on US, and ≥5 mm in maximum diameter. Inclusion also required availability of both US video recordings and post-therapy whole-body scintigraphy (WBS) images for assessment of RAI uptake, and at least 3 months of follow-up to evaluate treatment response before considering reoperation.

Patients with follicular or oncocytic carcinoma, poorly differentiated thyroid carcinoma, or differentiated high-grade thyroid carcinoma were excluded. Patients with distant metastases were also excluded because radioiodine uptake by distant lesions could alter biodistribution and potentially reduce the radiation dose delivered to cervical metastatic lymph nodes. This restriction also ensured that treatment response could be assessed objectively using high-resolution neck US. Lymph nodes <5 mm were excluded because reliable histopathologic correlation could not be established despite suspicious US findings.

No age-based exclusion criteria were applied, and adolescent patients were eligible for inclusion. Elevated anti-Tg antibody levels were not considered an exclusion criterion. In anti-Tg–positive patients, treatment response was assessed using structural imaging findings together with antibody trends, as described below. A history of non-thyroid malignancy was not considered an exclusion criterion because treatment outcomes were evaluated using thyroid cancer–specific structural and biochemical endpoints.

### Imaging and treatment protocol

For each lymph node, location (central vs. lateral), size (largest diameter on US), US features—including cortical hyperechogenicity (hereafter referred to as thyroidization), vascular pattern, composition, and the presence of punctate echogenic foci—and RAI uptake status were recorded. Cross-sectional imaging and FDG PET/CT were obtained selectively, as clinically indicated.

All ultrasound examinations were performed by the same sonographer (SI), who has more than 30 years of experience in thyroid and neck ultrasonography. Metastatic lymph nodes were identified using a combination of cytologic, biochemical, and sonographic criteria. Archived ultrasound video recordings were routinely reviewed during follow-up to facilitate direct comparison with prior examinations and maximize consistency of serial measurements.

All patients were prepared with a 3-week low-iodine diet and levothyroxine withdrawal to achieve TSH levels >30 mIU/L. Stimulated Tg and anti-Tg antibody levels were obtained before RAI therapy. No diagnostic WBS was performed before treatment. The mean interval between surgery and RAI administration was 2.5 months. The median administered RAI activity was 5550 MBq (range, 1110–5550 MBq). Administered activities were selected empirically according to disease extent, nodal burden, and physician judgment within the institutional treatment protocol. Patient-specific dosimetry was not performed.

Post-therapy WBS was performed on day 5 using a dual-head SPECT camera, with additional SPECT/CT imaging available in five patients. RAI status was determined by consensus after correlation of US video recordings and post-therapy WBS images by two experienced nuclear medicine physicians with expertise in both imaging technique. RAI-avid nodes were defined as clearly delineated lesions in lateral or central compartments. In cases with intense thyroid remnant activity where differentiation between remnant tissue and metastatic lymph node was not possible, the central compartment was categorized as “RAI-uncertain.”

### Response evaluation

Treatment response was assessed both on a by-node and by-patient basis. On the nodal level, a complete response was defined as disappearance or regression of the metastatic lymph node to barely visible size on US, with non-stimulated Tg <0.2 ng/mL in anti-Tg negative patients; in anti-Tg positive patients, normalization of antibody levels was additionally required. These patients were included in the follow-up program according to their new risk category. Partial response was defined as a ≥30% reduction in the largest nodal diameter measured on US. Volumetric measurements were not used. This threshold was selected to minimize the potential influence of measurement variability and to avoid classifying minor size differences as treatment response. Non-response was defined as stability, progression, or absence of RAI avidity. For statistical analysis, complete and partial responders were grouped together.

At the patient level, response was defined as complete structural resolution of nodal disease accompanied by biochemical remission. All other patients, including those demonstrating partial structural response, were classified as non-responders, as they continued to have persistent nodal disease, required ongoing T4-suppressive therapy, and remained potential candidates for future surgical intervention.

### Surgical management and pathology

RAI-non-avid and/or FDG-positive nodes were referred for surgical resection after at least 3 months of follow-up. Active surveillance was offered to patients with small RAI-avid or non-avid nodal metastases, that showed no objective response or only a partial response, until structural or biochemical progression. Nodal mapping with US was repeated before surgery. In selected patients, radioguided occult lesion localization was performed at the discretion of the surgical team to facilitate intraoperative identification of metastatic lymph nodes as reported before (23).

Risk categories were assigned according to the ATA-2025 stratification based on initial histopathology: 5 patients were classified as low-intermediate, 54 as high-intermediate, and 12 as high-risk. All high-risk patients were categorized as such due to large-volume (≥3 cm) nodal metastases, and none had distant metastases at diagnosis.

Aggressive subtypes of PTC were defined as tall cell, hobnail, columnar and diffuse sclerosing PTC (DSPTC), whereas the remaining subtypes were grouped as indolent. In patients directed to surgery because of treatment failure, histopathological examination of resected specimens included evaluation of necrosis and morphologic subtypes within nodal deposits. The presence of aggressive variants was recorded in the primary tumor and lymph nodes, even when they comprised < 30% of the primary tumor.

### Statistical analyses

Statistical analyses were performed with SPSS version 26 (IBM Corp, NY, USA). Shapiro–Wilk test was used to evaluate the distribution of the data. Student t test was used for normally distributed data. Non‐normally distributed data were analyzed with Mann-Whitney U test. Categorical data were presented as counts and percentages. Values were expressed as mean ± standard deviation or median, as appropriate. Categorical data were compared using the chi-square Fisher exact test. P < 0.05 was considered statistically significant.

## Results

### Study population

A total of 146 metastatic lymph nodes from 71 patients met the inclusion criteria. Baseline demographics, histopathologic features, RAI uptake status, and biochemical and US findings are summarized in **Tables 1**–**3**.

**Table 1:** Demographic, histopathologic and other related features of patients according to response status.

Characteristic of patients (n=71)	Responders (n=8)	Non-responders (n=63)	P value
Median age (year)	39 (23-59)	32 (13-71)	0.669
Sex (F/M)	6/2	35/28	0.294
Histopathology at first surgery			
Tumor size (mm)	22.2±25.8	20.9±14.0	0.604
Multifocality (Y/N)	1/7	37/26	**0.014**
Aggressive subtype (Y/N)	1/7	10/53	0.804
Aggressive subtype <30% (Y/N)	1/7	23/40	0.176
Maximum size of nodal metastasis (mm)	19.3±18.9	17.0±13.8	0.862
Number of metastatic lymph node	8.7±7.9	10.1±8.7	0.682
Existence of ENE (Y/N/U)	4/3/1	32/23/8	0.050
Existence of LVI (Y/N/U)	6/1/1	50/6/7	0.955
Stimulated Tg level (ng/mL)*	30.2±29.2	66.8±116.1	1.0
Existence of chronic thyroiditis (Y/N)	3/5	17/46	0.533
Anti-Tg (Positive/Negative)	2/6	15/48	0.941
RAI activity MBq (median)	5550 (1110-5550)	5550 (1110-7400)	0.467

F, Female; M, Male; Y/N/U, Yes/No/Unknown; Aggressive subtypes: tall cell, hobnail, columnar or DSPTC; ENE, extranodal extension; LVI, lymphovascular invasion; *: Anti-Tg positive patients were excluded. Bold values denote statistical significance. Multifocality was the only variable significantly associated with treatment outcome and was more common in non-responders.

**Table 2:** Lymph node-based evaluation according to RAI status, histopathology, location, size and US features.

Characteristic of lymph nodes (n=146)	RAI-avid (n=39, 27%)	RAI-non-avid (n=107, 73%)	P value
Lymph node response			**<0.0001**
Complete or partial	23	–	
No response	16	107	
Aggressive subtype (Y/N)	7/32	18/89	0.626
Aggressive subtype <30% (Y/N)	16/23	47/60	0.754
Location of mLN (Central/Lateral)	18/21	71/36	0.027
Size of mLN (mm)	11.0±4.8	10.2±4.6	0.319
US features			
Thyroidization (Y/N)	22/17	35/72	**0.009**
Existence of PEF (Y/N)	23/16	54/53	0.362
Increased vascularity (Y/N)	33/6	84/23	0.413
Cystic degeneration (Y/N)	32/7	75/32	0.324
FDG status (if available) (+/-)	7/4	40/14	NA

RAI, radioiodine; Y/N, Yes/No; PEF, punctate echogenic foci; mLN, metastatic lymph node; US, Ultrasonography; NA, not applicable. At the patient level, response was defined as complete structural resolution of nodal disease accompanied by biochemical remission. All other patients, including those demonstrating partial structural response, were classified as non-responders for analyses. Bold values denote statistical significance.

**Table 3:** Analysis of RAI-avid lymph nodes according to size, US features and RAI activity.

RAI-avid lymph nodes (n=39)	Responders* (n=23, 59%)	Non-responders (n=16, 41%)	P value
Size of mLN (mm)	11.0±4.8	10.8±5.0	0.989
US features			
Thyroidization (Y/N)	12/11	10/6	0.522
Existence of PEF (Y/N)	16/7	7/9	0.107
Increased vascularity (Y/N)	17/6	16/0	**0.026**
Cystic degeneration (Y/N)	6/17	1/15	0.112
RAI activity MBq (median)	5550(1110-5550)	5550(1110-5550)	0.823

*Lymph nodes showing complete (n=15) or partial (n=8) response was considered responders for statistical analyses. RAI, radioiodine; Y/N, Yes/No; PEF, punctate echogenic foci.Bold values denote statistical significance.

### Lymph node–based outcomes

**Figure 2:**
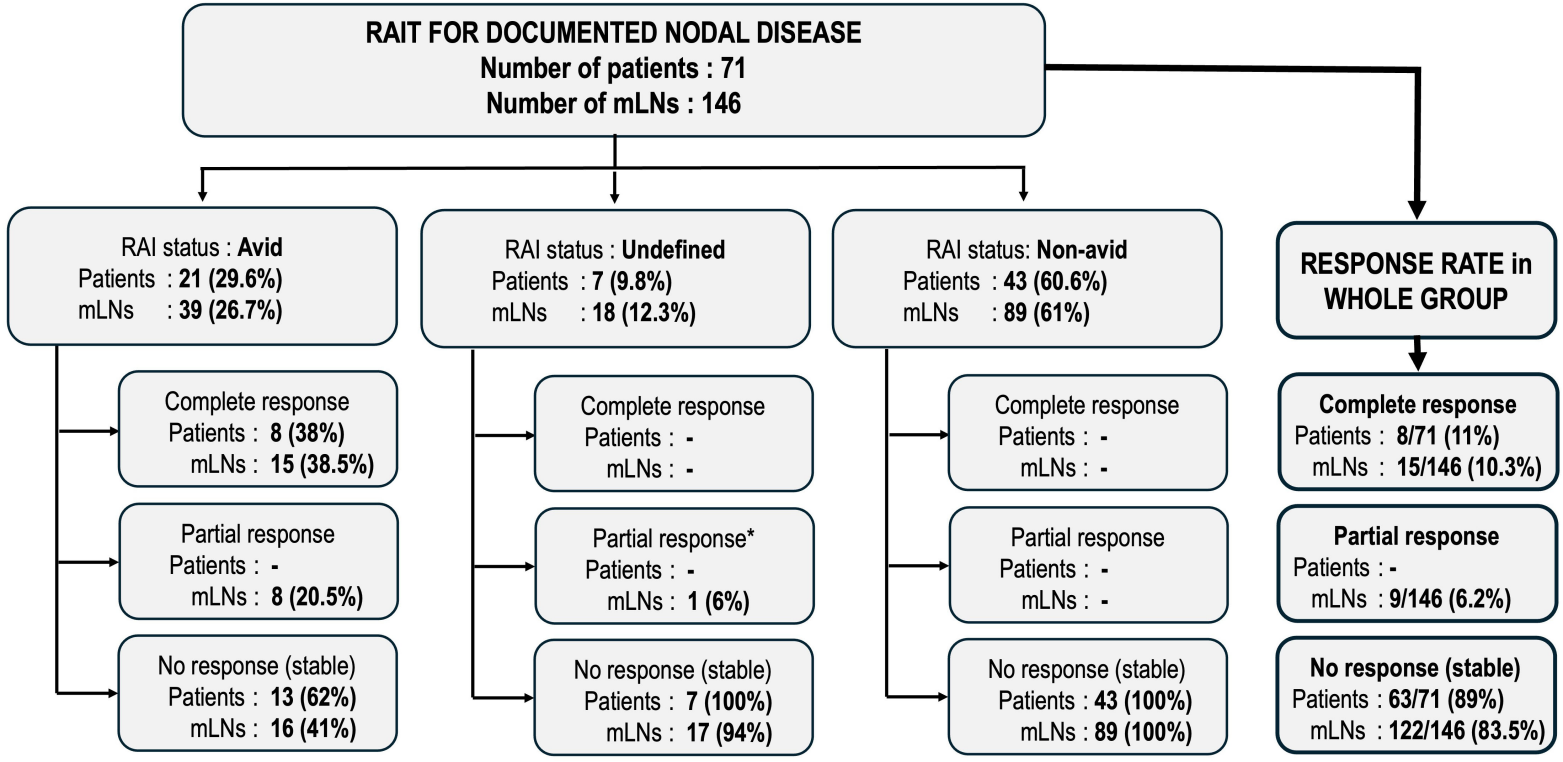
Flow diagram summarizing outcomes of RAI therapy for structurally documented residual nodal metastases in PTC. The cohort included 71 patients with 146 metastatic lymph nodes. The right panel summarizes overall response rates in the entire cohort. mLN, metastatic lymph node.

Among the 146 metastatic lymph nodes evaluated, 39 (26.7%) were RAI-avid and 89 (61%) were RAI-non-avid, while RAI status could not be objectively assessed (RAI-uncertain) in 18 (12.3%) central compartment nodes due to remnant interference. The distribution of RAI status and corresponding treatment responses at both the lymph node and patient levels are summarized in **Figure 2**.

Complete treatment response was observed in 15 of 39 (38.5%) RAI-avid metastatic nodes, and partial response in 8 (20.5%), yielding an objective response rate of 59.0%. No objective response was detected in 16 (41%) RAI-avid nodes. The largest metastatic node achieving a complete response measured 15 mm in greatest diameter. When all 146 metastatic lymph nodes were considered, complete response was observed in 10.3%, partial response in 6.2%, and no objective response in 83.5%. None of the RAI-non-avid metastatic lymph nodes demonstrated a partial or complete response during follow-up, indicating absence of measurable therapeutic effect in this subgroup.

**Figure 3:**
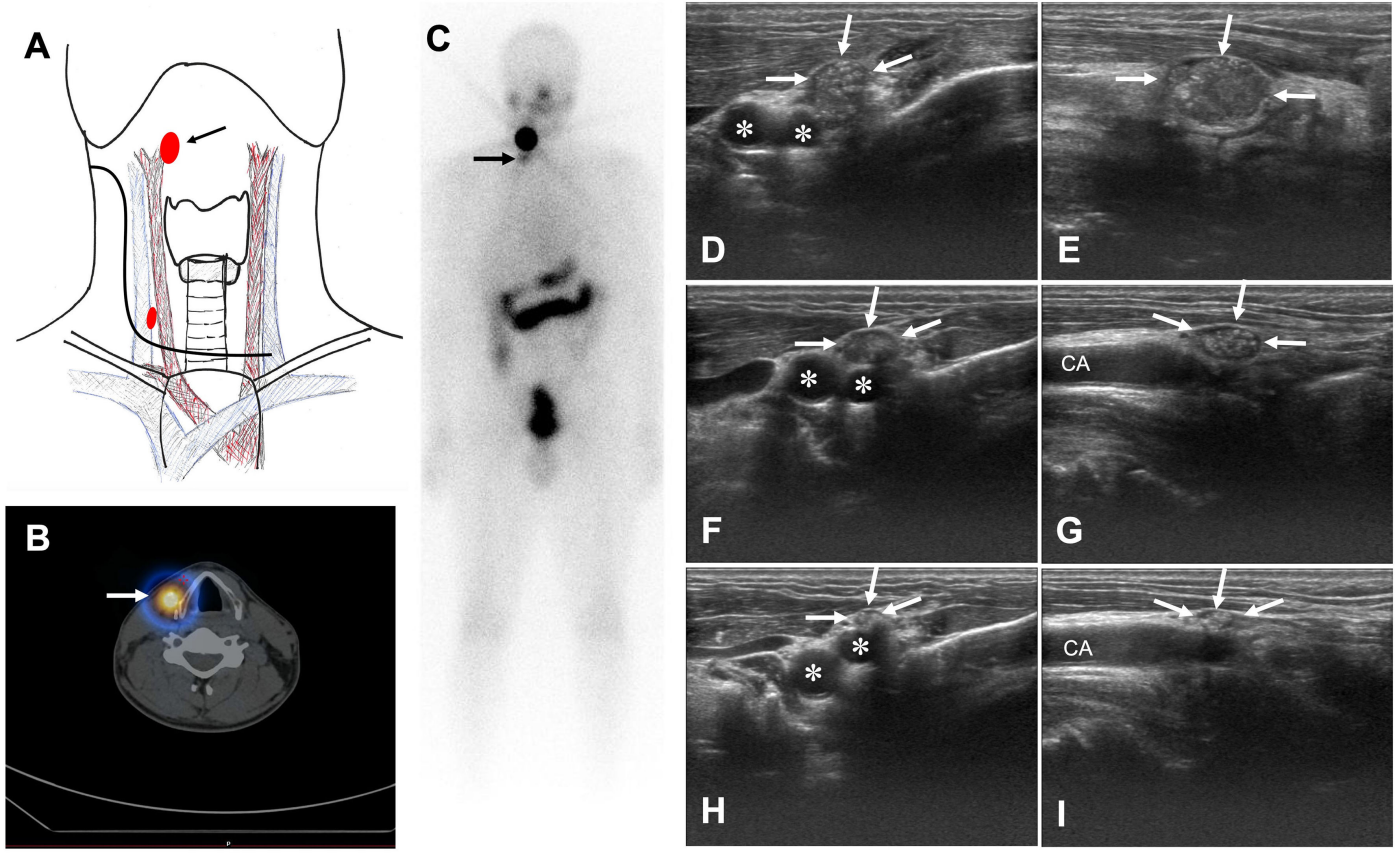
A 39-year-old man with ATA-2025 high-risk PTC (pT2N1bM0), underwent total thyroidectomy with central and right lateral lymph node dissection. Neck mapping at 6^th^ week showed residual metastatic lymph nodes **(A)**, including a 1.5-cm node (black arrow) and 0.9-cm node in the right lateral cervical chain. After 5550 MBq RAI, SPECT/CT **(B)** showed intense focal uptake in the dominant node (white arrow), and WBS **(C)** showed discernible uptake in the smaller node inferior to the dominant lesion (black arrow). Pretreatment US of the dominant node (white arrows) demonstrated thyroidization and punctate echogenic foci [**(D)**, axial; **(E)**, longitudinal]. Marked regression was seen 4 months after RAI [**(F)**, axial; **(G)**, longitudinal], and the node was barely visible at 1 year [**(H)**, axial; **(I)**, longitudinal]. The patient has remained in the excellent response category since August 2020. ✽, external and internal branches of the common carotid artery; CA, common carotid artery.

**Figure 4:**
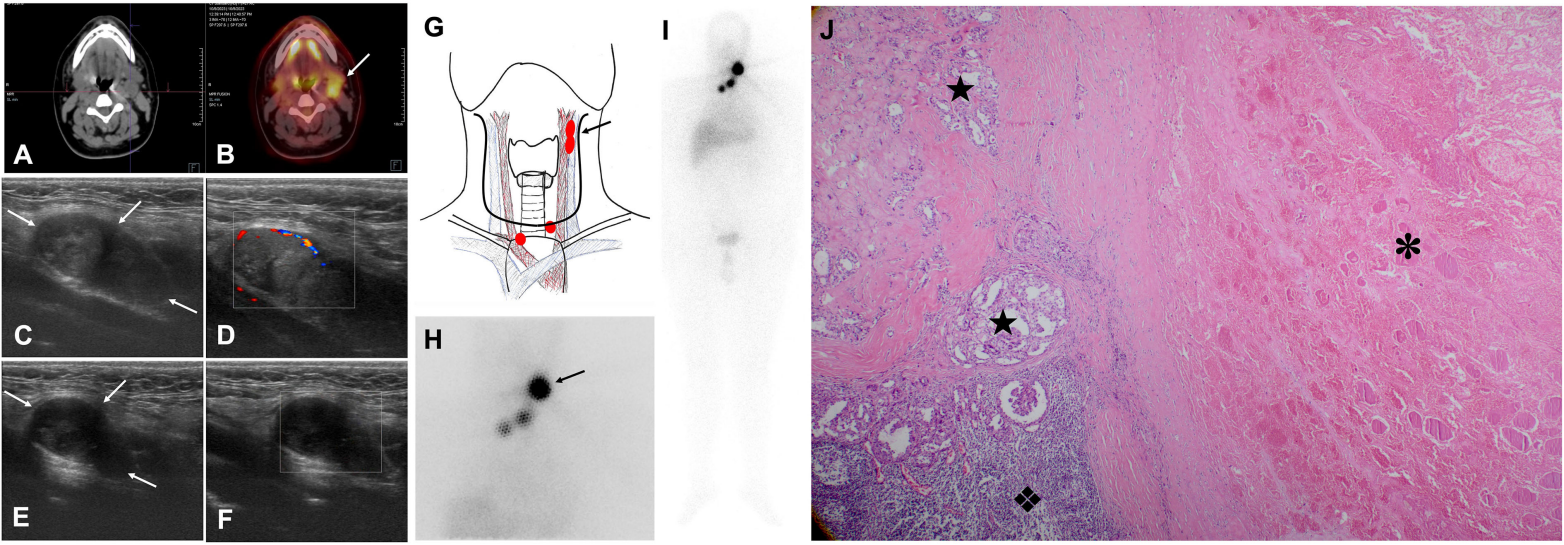
A 24-year-old man with ATA-2025 high-risk PTC (pT3N1bM0), underwent total thyroidectomy with central and bilateral cervical lymph node dissection. Residual metastatic lymph nodes were identified at 6 weeks in the left lateral and central compartments **(G)**. FDG PET showed uptake in the dominant 29×15 mm node **(A, B)**, and additional smaller central nodes (not shown). On pretreatment US, the dominant node (white arrows) demonstrated thyroidization **(C)** and increased peripheral vascularity **(D)**. After 5550 MBq RAI, the node decreased in size and became a hypoechoic **(E)**, and avascular **(F)** at 6 months. All lymph nodes were RAI-avid on planar neck imaging **(H)** and WBS **(I)**. Smaller central compartment nodes shown on the neck map resolved after therapy; however, reoperation was recommended for the partially responding FDG-positive node in the left lateral cervical region (black arrows). Histopathologic examination **(J)** revealed extensive necrosis (✽) involving >90% of the lymph node, with peripheral metastatic nests of follicular subtype (★) and preserved normal lymphoid tissue (❖) (hematoxylin–eosin stain, ×10).

**Figure 5:**
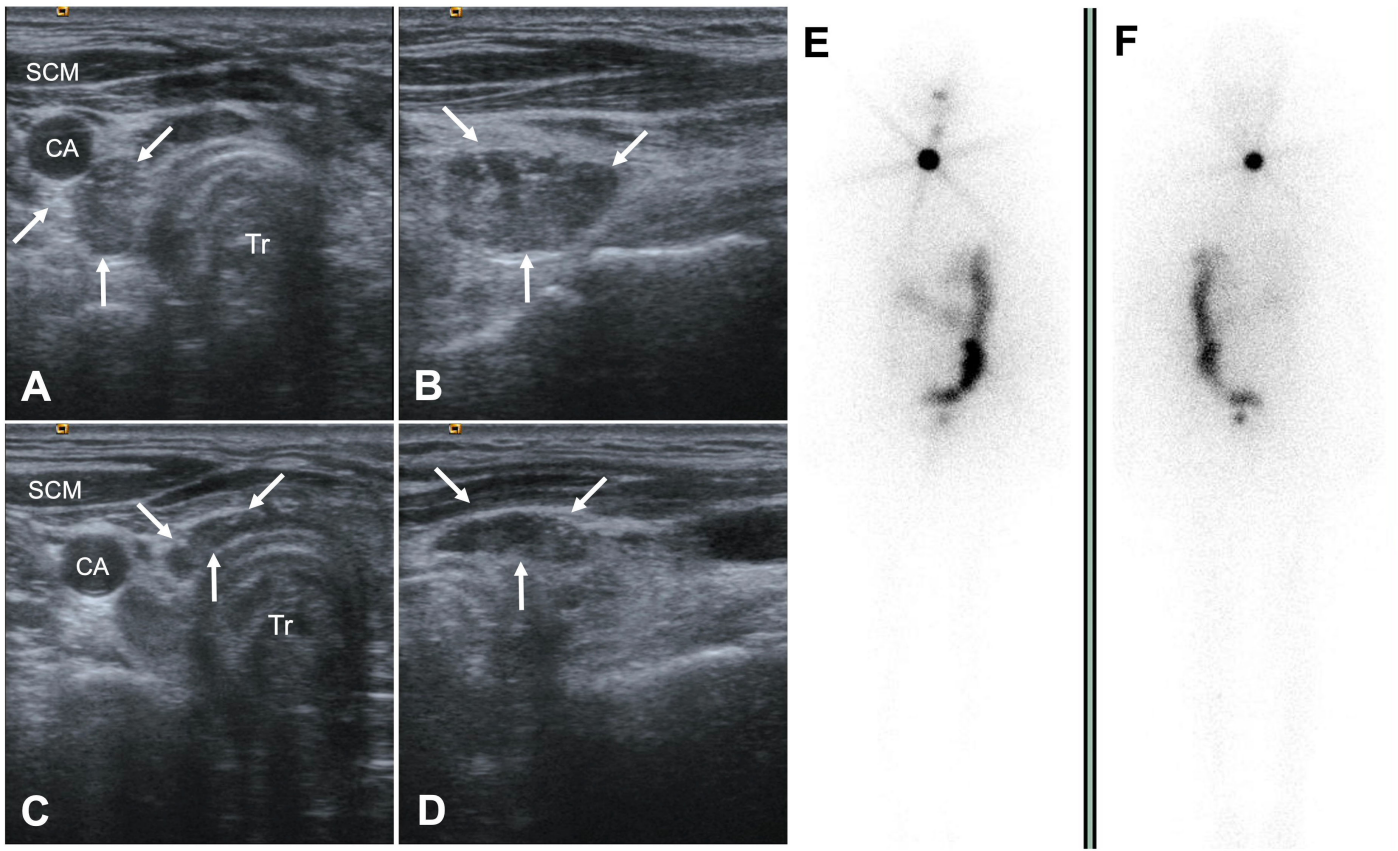
A 49-year-old woman with follicular subtype PTC, classified as ATA-2025 low-intermediate risk (pT1aN1aM0), underwent total thyroidectomy with central lymph node dissection. Residual metastatic lymph nodes (white arrows) were identified in the right paratracheal [**(A)**, axial; **(B)**, longitudinal] and pretracheal [**(C)**, axial; **(D)**, longitudinal] regions at 6 weeks postoperatively. The patient received 3700 MBq of RAI as initial therapy. Post-therapy WBS [**(E)**, anterior; **(F)**, posterior] demonstrated marked uptake corresponding to central compartment lymph nodes. Despite intense RAI uptake, follow-up US examinations showed no objective structural response, and reoperation was recommended 7 months after treatment due to rising anti-Tg antibody levels. Histopathologic evaluation revealed metastatic involvement in 2 of 5 resected lymph nodes (largest 1.5 cm), with morphology identical to the primary tumor. The patient has remained in the excellent response category since January 2020. SCM, sternocleidomastoid muscle; CA, common carotid artery; Tr, trachea.

**Figure 6:**
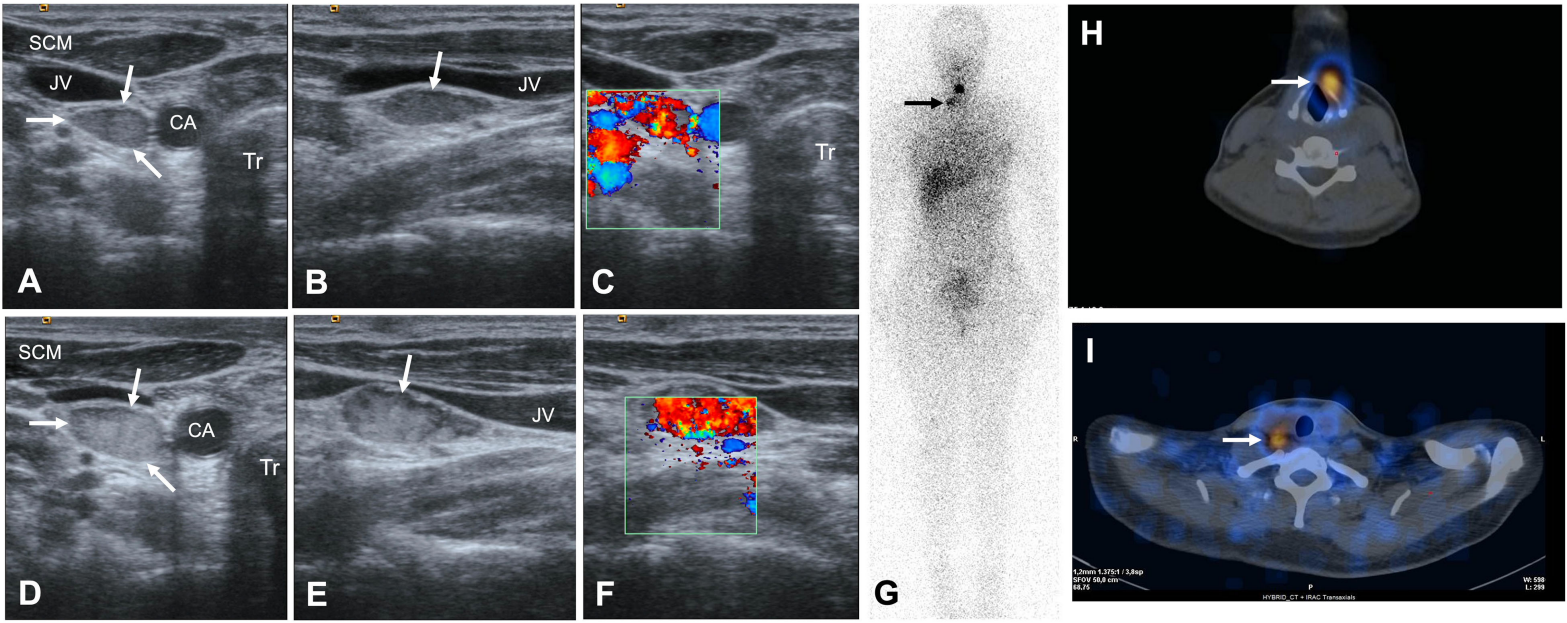
An 18-year-old woman with follicular subtype PTC, classified as ATA-2025 intermediate-high risk (pT1bN1bM0), underwent total thyroidectomy with central and right lateral lymph node dissection. Postoperative US **(A–C)** showed a metastatic node (white arrows) in lateral compartment, characterized by loss of the echogenic hilum, thyroidization [**(A)**, axial; **(B)**, longitudinal], and increased vascularity **(C)**, located posterior to the carotid artery and internal jugular vein. The patient received 3700 MBq of RAI as initial therapy. Anterior WBS **(G)** demonstrated mild focal uptake corresponding to the lateral metastatic node. SPECT/CT images showed remnant tissue activity around the thyroid cartilage **(H)** and confirmed mild uptake corresponding to the residual metastatic lymph node **(I)**. US performed on the day of post-therapy imaging demonstrated interval enlargement of the lymph node, likely related to inflammation and edema **(D, E)**, with persistent increased vascularity **(F)**. No objective structural response was observed during follow-up, and reoperation was recommended 11 months after RAI therapy due to rising Tg levels. Histopathologic examination revealed metastatic involvement in 1 of 9 resected lymph nodes (1.5 cm), with morphology similar to the primary tumor. The patient has remained in the excellent response category since October 2019. SCM, sternocleidomastoid muscle; CA, common carotid artery; JV, internal jugular vein; Tr, trachea.

**Figure 7:**
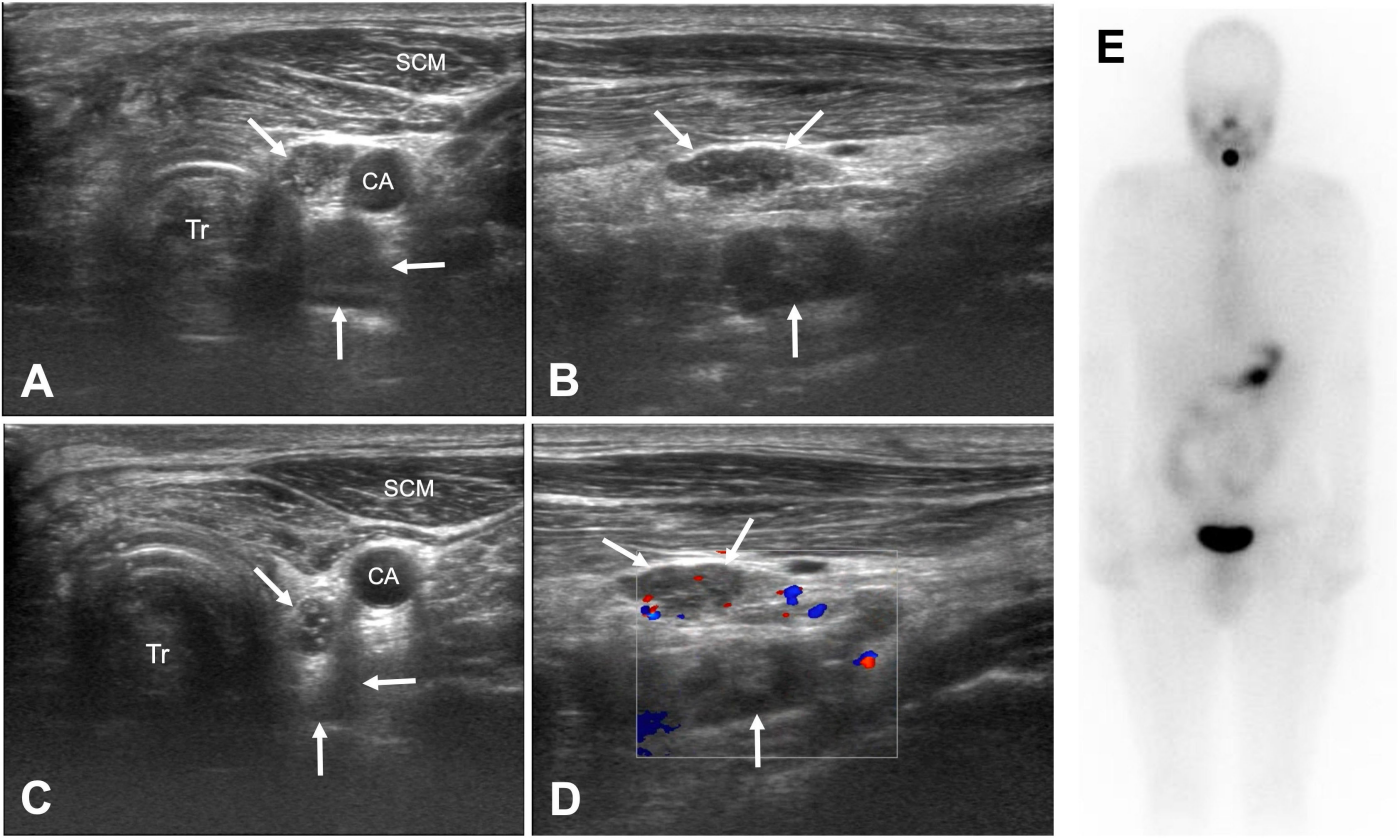
A 21-year-old male with mixed classical and follicular subtype PTC, classified as ATA-2025 intermediate-high risk (pT1bN1aM0), and Graves’ disease underwent total thyroidectomy with central lymph node dissection. Postoperative US demonstrated residual metastatic lymph nodes in both central compartments. Axial **(A)** and longitudinal **(B)** views show residual nodes (white arrows) in the left paratracheal region (right side not shown). The patient received 5550 MBq of RAI as initial therapy. Anterior WBS **(E)** demonstrated focal uptake in the midline consistent with thyroglossal duct activity, without evident uptake corresponding to residual lymph nodes. No objective structural response was observed on follow-up imaging. Axial US **(C)** showed persistent lymph nodes of similar size, and longitudinal Doppler imaging **(D)** demonstrated sustained increased vascularity. Reoperation was recommended 9 months after RAI therapy due to rising anti-Tg antibody levels. Histopathologic examination revealed metastatic involvement in 9 of 15 resected lymph nodes (largest 1.2 cm), with morphology similar to the primary tumor. The patient has remained in the excellent response category since August 2022. SCM, sternocleidomastoid muscle; CA, common carotid artery; JV, internal jugular vein; Tr, trachea.

RAI uptake status was strongly associated with treatment response (p<0.0001), whereas lymph-node size, location (central vs lateral), and administered RAI activity were not. **Figures 3**–**7** illustrate representative cases of residual nodal disease with varying RAI uptake status and treatment responses, including RAI-avid responders, RAI-avid non-responders, and RAI-non-avid disease.

### Patient-based outcomes

Among the 23 patients with RAI-avid metastatic lymph nodes, complete response was achieved in 8 (34.7%). In the overall cohort, the complete response rate at the patient level was 11% (8/71). All responders remained in the excellent response category at last follow-up, with a median follow-up of 77 months (range, 32–156 months), supporting the durability of treatment response.

### Clinical, pathologic and sonographic predictors of treatment response

Age, sex distribution, stimulated Tg levels prior to RAI, anti-Tg positivity, and administered RAI activity were not significantly different between responders and non-responders. Among histopathologic variables assessed at initial surgery—including tumor size, multifocality, presence of aggressive subtypes, maximal nodal size, extranodal extension, and lymphovascular invasion—only multifocality showed a statistically significant association with treatment outcome and was more frequent among non-responders.

Among the 23 patients with RAI-avid nodal disease, none had tall cell or hobnail subtype of PTC, although four had DSPTC, including one complete responder.

Among 48 patients with RAI-non-avid disease, seven had aggressive subtypes (three tall cell, three DSPTC, and one hobnail), and 17 had <30% aggressive components. The proportion of aggressive PTC subtypes (including <30% components) among non-responders was 36.5% (23/63). Of the seven DSPTC cases overall, only one of the three RAI-avid patients achieved a complete response, whereas the remaining six failed to respond.

Notably, the majority of RAI-non-avid tumors corresponded to histologically indolent subtypes rather than aggressive variants (26 classical, 5 infiltrative follicular, 1 solid, and 9 mixed subtypes).

Thyroidization was more frequent in RAI-avid than non-avid metastatic lymph nodes (p=0.009), whereas increased vascularity in RAI-avid nodal disease was associated with non-response (p=0.026).

### Outcomes after reoperation

Thirty-eight non-responding patients underwent reoperation after a mean follow-up of 16.3 months. Postoperatively, 22 patients (58%) achieved an excellent response, whereas 10 (26%) and 6 (16%) had biochemical and structural incomplete responses, respectively. Among those with structural incomplete response, two had the tall cell subtype and one had DSPTC; one patient with the tall cell subtype subsequently developed lung metastases during follow-up.

Extensive necrosis (>90%) was observed in a single RAI-avid patient with a large metastatic node (2.9 cm) who underwent surgery for incomplete response six months after RAI. In this patient, two smaller RAI-avid central metastatic lymph nodes disappeared completely, while the dominant node demonstrated partial regression in size and FDG positivity, prompting surgery. Retrospective US review revealed marked changes in echogenicity and vascularity, with transition from thyroidization and increased vascularity at baseline to an avascular, hypoechoic appearance after RAI (**Figure 4**). No additional necrotic nodes were identified among RAI-avid or RAI-non-avid metastases undergoing reoperation.

The remaining 25 non-responding patients (of 63 total non-responders) were managed with active surveillance at the time of analysis, with a mean follow-up duration of 34.1 months.

## Discussion

### Clinical context and rationale

Cervical lymph node metastases are common in PTC and are a major contributor to persistent or recurrent structural disease after initial therapy (24). Although RAI therapy is commonly used for eradication of presumed residual nodal disease, practice patterns vary substantially across centers, with the greatest disagreement occurring in patients with intermediate-risk PTC. This variability reflects the limited evidence regarding its true efficacy for nodal metastases and the difficulty of identifying patients most likely to benefit.

In routine practice, many patients referred for reoperation have previously received RAI therapy; however, without objective baseline documentation of nodal burden, treatment failure cannot be reliably inferred. Similarly, reports of high cure rates in RAI-avid nodal metastases often exclude non-avid disease by design and therefore do not reflect real-world effectiveness. These limitations underscore the need to evaluate RAI therapy in RAI-naïve patients with structurally documented nodal metastases. Unlike prior studies, our cohort was defined by structurally confirmed cervical lymph node metastases before initial RAI administration, thereby allowing true lesion-level response assessment.

### Methodological limitations of prior evidence

**Figure 8:**
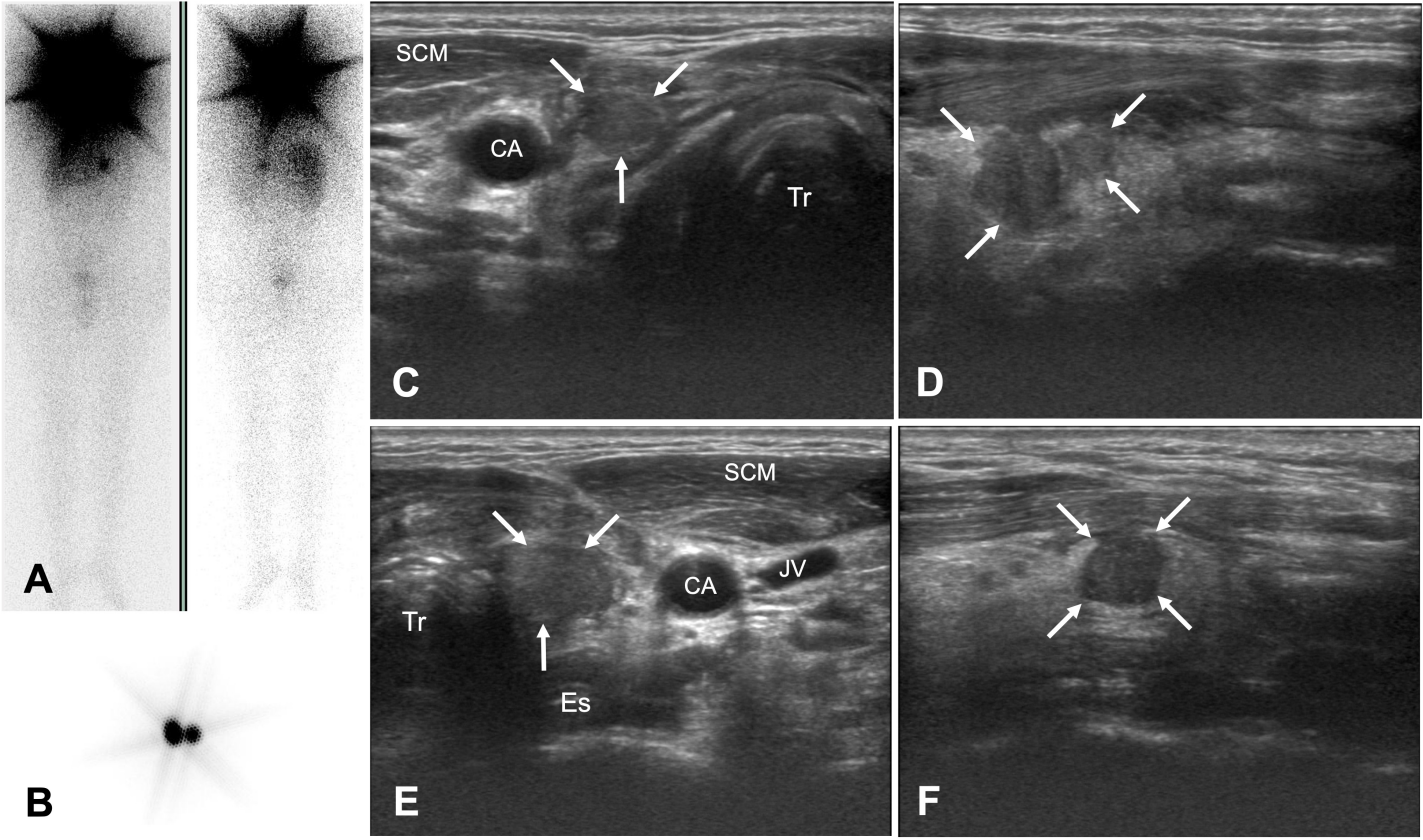
A 37-year-old man with follicular subtype PTC (T2NxM0) had a normal postoperative neck US at the sixth week, but remnant ablation with 1850 MBq was recommended because non-stimulated Tg measured was 5.8 ng/mL. Post-treatment WBS after 5 days showed intense RAI uptake in both thyroid beds causing star artifact on WB scan. Stimulated Tg level was measured 17.1 ng/mL before RAI therapy. US control on the day of WBS showed edematous lobulated remnant tissues (white arrows) in both thyroid bed which were invisible on pretreatment scan. **(C, E)** Axial and **(D, F)** longitudinal US images show hypoechoic remnant tissues in right and left thyroid beds, respectively. **(A)** anterior and posterior WB images and **(B)** planar neck images show prominent activity in thyroid bed. Described hypoechoic edematous remnants had completely disappeared on US exam performed 3 months after therapy, and serum Tg dropped to <0.04 ng/mL. SCM, sternocleidomastoid muscle; CA, common carotid artery; JV, jugular vein; Es, esophagus; Tr, trachea. .

Interpretation of the existing literature reporting relatively high treatment efficacy (5–14) is hindered by several major methodological limitations. First, nodal metastases were frequently diagnosed based on post-treatment WBS without systematic histopathologic confirmation. In the central compartment, physiological uptake from thyroid remnants —often accentuated by post-RAI inflammation and edema—may mimic metastatic lymph nodes on SPECT/CT, leading to overestimation of treatment response (**Figure 8**). Accurate recognition of this phenomenon would require correlation of pre-RAI neck US with repeat US performed on the day of post-treatment WBS; however, such imaging is generally avoided because of radiation safety considerations. Even in the lateral compartment, RAI accumulation alone should be interpreted cautiously, as false-positive uptake has been reported in sarcoidosis (25) and in a variety of benign and malignant conditions, including parasitic or ectopic thyroid tissue, pyramidal lobe remnants, and thyroglossal duct remnants, all of which may mimic metastatic lymph nodes (26).

Second, treatment response was commonly defined by disappearance of uptake on diagnostic WBS, which is less sensitive than post-treatment WBS. Even in the setting of optimal methodology, the reported response rates apply only to RAI-avid metastases and provide no information regarding RAI-non-avid nodal disease. However, rational use of RAI also requires knowledge of the prevalence of RAI-non-avid disease and factors influencing treatment success.

Third, study populations were often heterogeneous, including patients with distant metastases and mixed histopathologic tumor types, both of which may confound interpretation of treatment outcomes. In patients with distant metastases, RAI uptake by distant metastasis may alter biodistribution and reduce the radiation dose delivered to metastatic lymph nodes, thereby complicating assessment of nodal treatment efficacy.

### Prevalence and clinical impact of RAI-non-avid nodal disease

This study provides protocol-based observational evidence regarding the effectiveness of RAI therapy for structurally documented residual cervical nodal metastases in RAI-naïve patients with PTC. To our knowledge, it is among the first studies to demonstrate that a considerable proportion of metastatic lymph nodes (61%) from PTC are RAI-non-avid. Because RAI status could not be objectively determined in an additional 12.3% of central metastatic nodes (RAI-uncertain) due to remnant interference, the true prevalence of RAI-non-avid disease may be higher and could approach 70%.

The objective treatment response was restricted to RAI-avid metastatic nodes; no RAI-non-avid metastasis demonstrated regression during follow-up. These findings indicate that RAI non-avidity is a major determinant of persistent nodal disease. Accordingly, the overall complete response rate across all lymph nodes—predominantly composed of RAI-non-avid disease—was low (10.3%), providing a plausible explanation for the high rates of persistent nodal disease observed in PTC patients treated with adjuvant RAI therapy.

This observation aligns with the seminal autoradiographic work of Fitzgerald et al. (1949), which demonstrated iodine uptake in only 28% of PTC and revealed substantial intratumoral heterogeneity (27). The rigorous autoradiographic methodology used in that study provided direct tissue-level evidence of iodine handling. Reported intratumoral heterogeneity may partly account for the limited treatment response observed even among RAI-avid metastatic nodes, as regional differences in iodine handling could reduce effective radiation delivery to all tumor cells. Remarkably, the proportion of RAI-avid patients in our cohort (32%) closely mirrors these historical data, underscoring that limited RAI-avidity is not a modern phenomenon but an intrinsic biological property of many PTC types.

### Incomplete response in RAI-avid disease

The complete response rate among RAI-avid metastatic lymph nodes in the present cohort (38.5%) was lower than the 76–96% reported in prior series (5–14), most likely reflecting the use of objective structural response criteria rather than disappearance of uptake on diagnostic WBS. Even when partial responses were included, the overall response rate for RAI-avid nodal disease (59%) remained below those previously reported. Escalation of RAI frequency or administered activity commonly reflects an attempt to counterbalance increasing clinical and histopathologic risk, yet our data indicate that this strategy may not meaningfully change the course of nodal disease in many patients. Nevertheless, in the subset of patients who achieve a complete response, remission appears durable over long-term follow-up.

Although it is well recognized that administration of fixed RAI activities results in substantial interpatient variability in the absorbed radiation dose delivered to metastatic lymph nodes, patient-specific dosimetry was not feasible in our cohort because of practical limitations in routine implementation and reimbursement constraints. Importantly, when achievement of dosimetrically calculated target doses would require administration of very high RAI activities, the clinical rationale for escalating treatment beyond commonly used empiric activities to manage nodal disease becomes questionable, particularly when surgical resection remains a definitive alternative.

The study by Maxon et al., which remains one of the most frequently cited references supporting dosimetry-guided treatment of nodal metastases, recommended an absorbed dose >80 Gy for eradication of metastatic nodal disease (28, 29). In that series, nodal mass was estimated from anterior 1:1 rectilinear neck scans, assuming spherical or ellipsoidal geometry, an approach now considered technically outdated. Nevertheless, the authors reported favorable outcomes in 74% of patients and 81% of nodal metastases after a first treatment delivering >85 Gy, achieved with administered activities >3700 MBq. In a subgroup with isolated nodal metastases, similar administered activities were estimated to deliver >140 Gy, with reported success rates of 86% at the patient level and 90% at the nodal level.

In contrast, we did not observe comparable response rates among RAI-avid nodal metastases treated with similar empiric activities. This discrepancy most likely reflects differences in response definition, as our study relied on objective structural criteria rather than scintigraphic disappearance of uptake. Moreover, no clear association was identified between administered RAI activity and treatment outcome in our cohort. These findings do not negate a potential role for dosimetry-guided therapy but highlight that uptake alone is insufficient to predict response.

### Histologic subtype and the paradox of indolent non-avid disease

Previous studies have similarly demonstrated that aggressive variants such as tall-cell and hobnail PTC are frequently associated with adverse clinicopathologic features, reduced RAI-avidity, and poorer treatment outcomes, supporting the concept that histologic subtype remains an important modifier of therapeutic responsiveness despite substantial biological heterogeneity within these categories (30).

Contrary to expectations, most RAI-non-avid metastases originated from histologically so-called indolent tumor subtypes. The proportion of aggressive PTC subtypes did not differ significantly between responders and non-responders or between RAI-avid and RAI-non-avid groups. The absence of a statistically significant association may reflect the limited sample size or the unexpectedly high prevalence of RAI-non-avid disease among tumors otherwise classified as indolent.

Although aggressive PTC subtypes have been associated with reduced RAI-avidity, histologic features alone did not fully account for treatment response in the current study. A considerable proportion of RAI-non-avid metastases arose from tumors classified as indolent subtypes, suggesting that loss of RAI-avidity may occur independently of classic high-risk histopathologic features.

Although RAI uptake is generally linked to tumor differentiation, the presence of RAI-non-avid metastatic nodal disease does not always indicate poor prognosis in patients with PTC. In clinical practice, a significant number of RAI-non-avid PTC appears to exhibit less aggressive biological behavior compared to aggressive subtypes. It may therefore be hypothesized that patients with recurrent nodal disease who remain under active surveillance over long follow-up periods without significant structural or biochemical progression represent this subgroup with RAI-non-avid nodal disease. In support of this concept, salvage surgery resulted in an excellent treatment response in 58% of RAI non-responders in our cohort. Notably, three out of 6 patients in the structural incomplete response category after salvage surgery had aggressive subtypes, further underscoring the distinct biological behavior of aggressive PTC subtypes.

In our cohort, tall cell and hobnail variants of PTC were uniformly RAI-non-avid, suggesting that current RAI administration strategies in this subgroup warrant reconsideration, as patients with such tumors often receive more frequent and higher RAI activities despite limited evidence of therapeutic benefit. In contrast, DSPTC demonstrated RAI avidity in some cases but rarely achieved complete response, indicating partial preservation of iodine-handling capacity.

### Impact of nodal metastasis size

Another important variable in evaluating RAI efficacy for nodal disease is the size of metastatic lymph node. Ilhan et al. reported a treatment success rate of 96% for nodes <0.9 mL, declining to 42% for those ≥0.9 mL; similarly, success rates were 94% and 57% for nodes with a short-axis diameter <1 cm and ≥1 cm, respectively (5). That cohort included histologically heterogeneous tumors and patients with distant metastases; nevertheless, nodal size emerged as the only variable associated with treatment efficacy among RAI-avid metastases.

The widely held view that smaller nodal metastases respond more effectively to RAI has provided a conceptual basis for the treatment of presumed micrometastatic disease. However, from a radiobiological perspective, a metastatic deposit must exceed a minimum size to absorb the full energy of beta emission. Spherical dosimetry models demonstrate that, even under ideal conditions of homogeneous intratumoral activity distribution, absorbed radiation dose declines markedly with decreasing tumor size (especially below 1 mm, which is roughly equal the size of two thyroid follicles or 100 thyrocytes) (31). Dose reductions are even more pronounced in worst-case scenarios, in which RAI activity is distributed nonhomogeneously or predominantly at the tumor periphery.

In this study, nodal size was not associated with treatment efficacy in either the overall cohort or the RAI-avid subgroup. Notably, the largest residual metastatic node in our series (29 mm) demonstrated near-complete necrosis, whereas other smaller RAI-avid nonresponding nodes showed no histopathologic evidence of necrosis. These observations suggest that nodal size alone is unlikely to be a critical determinant of response to RAI.

For readers less familiar with neck US, it is important to note that the size thresholds cited in the literature (0.9 mL in volume or a short-axis diameter of 1 cm) correspond to relatively large lymph nodes; in our cohort, only the largest metastatic node illustrated in **Figure 4** approached these limits. Although it is not possible to either prove or disprove that RAI treats unproven micrometastases, routine adjuvant RAI therapy—defined as administration based solely on histopathologic risk factors in the absence of structural or biochemical evidence of disease—should be applied cautiously, as it is unlikely to provide meaningful benefit for unselected patients who may already harbor residual macrometastatic disease.

### Implications for patient selection for RAI therapy

Although complete response rates in the RAI-avid group remained suboptimal, RAI may still represent a realistic alternative to high-risk reoperations, especially in centers with limited surgical expertise. The optimal number of RAI treatments and the optimal activity/dose for RAI-avid nodal disease remains unclear. Although current guidelines recommend compartment-based surgery for persistent nodal disease after initial treatment, repeating RAI therapy, for at least a group of RAI-avid nodal disease, may be considered after weighing the risks of reoperation against those associated with additional RAI.

Given the high prevalence of RAI-non-avid disease observed in this cohort, careful patient selection is essential when residual nodal metastases are identified after initial surgery in PTC. Two principal management pathways may be considered. The first involves functional imaging with iodine-based radiopharmaceuticals (I-123, I-131, or I-124) to confirm RAI avidity at the level of metastatic lymph nodes before administration of therapeutic activities. US features—particularly thyroidization—were more common in RAI-avid metastatic nodes and may assist in noninvasive prediction of RAI-avidity. Diagnostic WBS and, where appropriate, FDG PET may further refine selection of patients and justify the use of higher administered activities in selected cases. Conversely, absence of demonstrable uptake would argue against empiric RAI escalation and favor alternative strategies. Such an approach may improve therapeutic precision while reducing unnecessary exposure to higher RAI activities and associated short- and long-term toxicities.

In the RAI-non-avid subgroup, long-term outcomes appear to depend primarily on early detection and timely, well-planned curative surgery rather than escalation of RAI therapy. Nevertheless, even when nodal disease is confirmed to be RAI-non-avid or when patients harbor aggressive subtypes that are unlikely to respond to therapeutic RAI, remnant ablation may still have a role in patient management. In selected patients—particularly those with non-stimulated Tg >0.2 ng/mL—remnant ablation may enhance the sensitivity of biochemical surveillance and facilitate timely use of additional imaging to detect structural disease amenable to salvage surgery.

### Potential molecular explanations

The introduction and widespread adoption of the term “differentiated thyroid cancer” in the mid-20th century unintentionally reinforced a false equivalence: that morphologic differentiation implied preserved functional differentiation. This misinterpretation became deeply rooted in clinical practice, leading to the historically widespread empiric use of RAI therapy under the assumption that all differentiated thyroid cancers, including PTC, were metabolically RAI-avid. A substantial proportion of RAI-avid metastases in our cohort demonstrated no objective response, indicating that iodine uptake alone is insufficient for therapeutic success. These findings may represent a biologically distinct subset that could be conceptualized as “mis-differentiated” disease—tumors that retain partial iodine transport but appear to have impaired organification, retention, or intracellular handling (32).

From a radiobiologic perspective, absorbed dose—not administered activity—determines therapeutic efficacy. Even when uptake is measurable, lesion-specific biokinetics may yield absorbed doses far below cytotoxic thresholds. Our inability to identify an association between administered activity and response further supports the concept that dose delivery is governed by intrinsic tumor biology or microenvironmental factors rather than administered activity.

Although molecular profiling was not available in this cohort, the observed patterns of uptake and response may reflect underlying molecular mechanisms regulating iodine handling in PTC. These observations underscore the importance of investigating the molecular basis underlying differences between RAI-avid and RAI-non-avid metastases, as well as between RAI-avid responders and non-responders. Systematic molecular analyses of histopathologic specimens from real-world cohorts such as this may help clarify the biological determinants of RAI-avidity and therapeutic response.

### Strengths and limitations

This study has several limitations. First, the retrospective design introduces potential selection bias, although the dataset was derived from a prospectively followed cohort. Second, SPECT/CT could not be performed in all patients; however, assessment of RAI status and treatment response relied primarily on high-resolution neck US, which represents a reliable modality for evaluation of cervical lymph nodes. Third, the sample size, while modest, reflects the stringent selection criteria and the substantial clinical and logistical demands associated with comprehensive diagnostic evaluation, longitudinal follow-up, reoperative procedures, and detailed histopathologic and imaging correlations. In addition, molecular tumor profiling was not available, precluding assessment of potential associations between genetic alterations and RAI-avidity or treatment response. This limitation is particularly relevant because molecular characterization of metastatic nodal deposits remains limited in the literature and may provide important insights into mechanisms underlying RAI-avidity and treatment response.

The principal strength of this study lies in the integrated evaluation of US and post-treatment WBS by the same experts experienced in both modalities, thereby minimizing interobserver variability. Furthermore, the routine use of systematic neck mapping and the radioguided occult lesion localization technique during reoperations enabled objective histopathologic validation of US-detected metastatic nodes, strengthening the accuracy of imaging–pathology correlations.

## Conclusions and clinical implications

Clinically, these results favor a shift from morphology-based decision-making toward a theranostic framework integrating functional imaging, molecular insights, and—where feasible—lesion-level dosimetry.

RAI-non-avid nodal metastases emerged as a principal contributor to treatment failure, as most residual lesions lacked measurable iodine uptake and none demonstrated objective response. At the same time, RAI-avidity alone was insufficient to ensure therapeutic success, as a substantial proportion of RAI-avid metastases failed to regress, consistent with impaired iodine organification, retention, or effective dose delivery. Nevertheless, durable remission in responders indicates that meaningful benefit can be achieved when functional iodine metabolism is preserved.

Histologic aggressiveness and RAI responsiveness were not tightly coupled. Aggressive PTC subtypes were largely non-responsive to RAI despite occasional uptake, whereas many indolent subtypes demonstrated RAI non-avidity yet stable clinical behavior. Thus, RAI avidity should not be equated with favorable prognosis, nor non-avidity with aggressive disease.

Collectively, these findings support a more selective approach to RAI therapy in residual nodal disease. Confirmation of RAI-avidity—potentially using diagnostic iodine imaging—followed by individualized treatment selection may be more appropriate than routine empiric administration of high RAI activities. Emphasis should also be placed on optimal preoperative nodal assessment to minimize residual disease, given the limited efficacy of RAI for many nodal metastases and the risks associated with reoperative surgery. Such a strategy would also be expected to reduce the use of empiric adjuvant RAI, which is frequently administered to target presumed but undocumented nodal disease.

Ultimately, improving outcomes in nodal disease will depend less on increasing RAI activity and more on restoring or identifying functional iodine metabolism, optimizing preoperative nodal assessment, and selecting the appropriate modality—RAI, surgery, or surveillance—based on tumor biology rather than histology alone. The durability of RAI response seen in our cohort confirms that when functional differentiation is preserved or restored, RAI retains curative power. The challenge moving forward is not to abandon RAI but to apply it selectively and rationally, integrating molecular diagnostics, functional imaging, individualized dosimetry, and redifferentiation algorithms.

## Data Availability

The raw data supporting the conclusions of this article will be made available by the authors, without undue reservation.
